# The Human Microbiota in Endocrinology: Implications for Pathophysiology, Treatment, and Prognosis in Thyroid Diseases

**DOI:** 10.3389/fendo.2020.586529

**Published:** 2020-12-04

**Authors:** Giovanni Docimo, Angelo Cangiano, Roberto Maria Romano, Marcello Filograna Pignatelli, Chiara Offi, Vanda Amoresano Paglionico, Marilena Galdiero, Giovanna Donnarumma, Vincenzo Nigro, Daniela Esposito, Mario Rotondi, Giancarlo Candela, Daniela Pasquali

**Affiliations:** ^1^ Division of Thyroid Surgery, Department of Medical and Advanced Surgical Sciences, University of Campania “Luigi Vanvitelli”, School of Medicine, Naples, Italy; ^2^ Department of Advanced Medical and Surgical Sciences, University of Campania “Luigi Vanvitelli”, Naples, Italy; ^3^ Department of Experimental Medicine, University of Campania “Luigi Vanvitelli”, Naples, Italy; ^4^ Department of Precision Medicine, University of Campania ”Luigi Vanvitelli”, Naples, Italy; ^5^ Department of Endocrinology, Institute of Medicine, Sahlgrenska Academy, University of Gothenburg and Sahlgrenska University Hospital, Gothenburg, Sweden; ^6^ Istituti Clinici Scientifici Maugeri IRCCS, Unit of Internal Medicine and Endocrinology, Laboratory for Endocrine Disruptors, University of Pavia, Pavia, Italy

**Keywords:** ****gut microbiota, thyroid, cancer, autoimmunity, Hashimoto autoimmune thyroiditis

## Abstract

The human microbiota is an integral component in the maintenance of health and of the immune system. Microbiome-wide association studies have found numerous diseases associated to dysbiosis. Studies are needed to move beyond correlations and begin to address causation. Autoimmune thyroid diseases (ATD) are one of the most common organ-specific autoimmune disorders with an increasing prevalence, higher than 5% worldwide. Most frequent manifestations of ATD are Hashimoto’s thyroiditis and Graves’ disease. The exact etiology of ATD remains unknown. Until now it is not clear whether bacterial infections can trigger ATD or modulate the efficacy of treatment and prognosis. The aim of our review is to characterize the microbiota and in ATD and to evaluate the impact of dysbiosis on treatment and prognosis. Moreover, variation of gut microbiome has been associated with thyroid cancer and benign nodules. Here we will characterize the microbioma in benign thyroid nodules, and papillary thyroid cancer to evaluate their implications in the pathophysiology and progression.

## Gut Microbioma

A lot of microbes, like bacteria, fungi, archaea, viruses, and protozoa, colonize our body. Many of these reside within the gastrointestinal tract and moreover on the ocular surface (1); they are predominantly bacterial, and together these microbes collectively form the gut microbiota. Gut microbiota contains trillions of microorganisms, about 66% of the human microbiota, including at least one thousand different species of known bacteria with more than 3 million genes (much more times than human genes). Thirty-three percent of these is common to all people, while 66% are explicit to every single one of us. This microbial flora has been coevolute with human being in a symbiotic relationship for millennia and, thusly, it finds itself involved in many essential activities for our organism such as digestion and nutritional absorption (2–4), development of the host’s immunity system (3), and pathogenesis (5, 6). The gut microbiota was found to play an important role in maintaining the nutritional, metabolic, and immunological balance in the host (7). In addition to its intended role in maintaining gastrointestinal homeostasis, it also performs metabolic functions in digestion and nutrient absorption, detoxification, and vitamin synthesis. Furthermore, the microbiota also appears to be an important factor in the development of the lymphoid system, 70% of which resides in the intestinal mucosa. In the literature there are various examples, both on mouse models and on humans, which demonstrate an altered composition of the intestinal microbiota in a number of different pathological disorders, including obesity, compared to the “physiological” microbiota. For this reason, a substantial part of the studies on the gut microbiota treated so far has been dedicated to this topic. Interest in the role of the microbiota in health has increased, as evidenced by the May 2016 announcement of the National Microbiome Initiative (NMI) to promote the use of microbiota science in health care, but also for environmental restoration and food production. The new genome resource increases the capability to identify gut metagenomes over 87% (8, 9). A recent study by Huiting et al. showed that iodine treatment affected the modulation mechanisms of thyroid function and the gut microbiota in obese mice. Administration of KIO3 in these animals led to weight reduction, increased concentration of thyroid hormones, alteration in the expression of the genes involved in thyroid biosynthesis, and cause various effects on the gut microbiota, changing the composition of the intestinal microenvironment, resulting in an imbalance of gut microbes: increases in pathogenic bacteria (*Enterococcus*, *Clostridium*, *Fusobacterium nucleatum*, *Burkholderiales*, *Helicobacter*), decrease in beneficial ones (*Lactobacilli, Bifidobacteria*), quite the contrary to what has been observed in non-obese hosts (p < 0.05). Therefore, even with iodine dosages considered safe, obesity and quantitative and qualitative changes in the microbiota can increase the risk of thyroid dysfunction (10). Frohlich and Wahl concluded in their review that the composition of the microbiota and the different representation of its individual components in the various parts of the gastrointestinal system influence the absorption of I^−^, also modifying the enterohepatic circulation of thyroid hormones. Moreover, minerals such as Se, Fe, and Zn seem to be involved in the interaction between microbiota and host (11). The human microbiota seems to be a key regulator of health and diseases and the relevance of its influence on human health is gradually emerging. Gut dysbiosis is an alteration of the microbiota in its physiological function in the gastrointestinal tract; this condition can lead to local phlogosis and alteration of metabolic functions (12). In the gut dysbiosis there’s a low microbial diversity (13) that is related with an extended range of human diseases, including changes in host immune status (14), asthma, allergies, inflammatory bowel disease (15–18), irritable bowel syndrome (19), obesity (20), chronic kidney disease (21), cardiovascular disease (22), and changes in blood pressure regulation (23). Additionally, alterations in gut microbial composition or function have been associated with age-related health impairment (24, 25). However, it’s still difficult to establish the connections between the dysbiosis and these conditions, except for few cases. Furthermore, also changes in the viral component of microbiome (virome) can be associated with infectious and inflammatory diseases. Intestinal eukaryotic viruses have been implicated in triggering human type 1 diabetes mellitus (T1D). Changes in the intestinal virome, in particular Circoviridae-related sequences, have been found in who developed serum autoantibodies associated with T1D, suggesting that changes in the intestinal virome preceded autoimmunity (26). Some viruses establish subclinical lifelong persistent or latent infections in their host thereby becoming part of the normal microbiome. Human viral ecology is poorly understood yet. Paul G. Cantalupo et al. have developed a virus detection and discovery computational pipeline, Pickaxe, and applied it to NGS databases provided by The Cancer Genome Atlas (TCGA). They analyzed a collection of whole genome (WGS), exome (WXS), and RNA (RNA-Seq) sequencing libraries from 3,052 participants across 22 different cancers. HBV was also detected only in one sample of thyroid cancer, but these HBV sequences were due to sample cross contamination during sequencing, so they need to be confirmed. Moreover, viruses have long been considered potential triggers of autoimmune diseases (27).

## Gut Microbiome and Autoimmunity

The relation between autoimmunity and other systemic diseases has been for a long time examined by worldwide literature (28–30), moreover focusing on new therapeutic implications in both surgical and medical field (31–38). The pathogenesis of autoimmune disorders is due to genetic (4–7, 23–25, 39–41), stochastic (42), and environmental (43–49) factors. In the environmental factors that have gained attention during the last decade, there is the intestinal microbiota. Several studies have supported a connection of altered microbiota composition with the beginning of several different autoimmune disorders, suggesting its role in the pathogenesis. These include Type I diabetes (45, 49); rheumatoid arthritis (50–53); systemic lupus erythematous (54, 55); inflammatory bowel disease comprising Cohn’s disease and ulcerative colitis (19, 56); Bechet’s disease (57); autoimmune skin conditions including vitiligo (58), atopic dermatitis (59, 60); psoriasis vulgaris (61), and autoimmune neurological diseases (62, 63). About 5–10% of the variability of bacterial taxa ascertained among individuals should be explained by genetic. Among the bacterial taxa transmitted, most of these is linked to genes involved in innate immunity (64). It is known that gastrointestinal microbiota is in reciprocal relationship with the host’s immune system: immune system, through an equilibrium of pro- and anti-inflammatory pathways, has an important role in the modulation of the microbiome community; on the other hand microbiome has a critical role in the development of the immune system (65).

## Microbiome and Thyroid Autoimmune Diseases

Autoimmune thyroid disease (ATD) is a common autoimmune disorder. It’s an organ-specific disorder and its prevalence is higher than 5% worldwide and still increasing (66). The principal manifestation of the ATD are Graves’ Disease (GD) and Hashimoto’s Thyroiditis (HT). In HT there’s a destruction of the thyroid cells that causes hypothyroidism, often detected in a subclinical condition (67), while in GD autoantibodies bind thyreotropin receptors stimulating an excessive production of thyroid hormone leading to hyperthyroidism. The role of T-lymphocytes and their cytokines is indispensable for the immune modulation, but it’s complex and full of connections with other components of the immunity. The correlation between gastrointestinal microbiota and development and progression of ATD has not yet been fully clarified. Nowadays, the analysis of reciprocal influence between microbiome and HT is a topic of considerable interest in the literature, since HT is the most frequent autoimmune disorder worldwide. There are several studies, both in animal models and in human, suggesting a link between modification in microbiota with the origins and development of ATD showing its key role in the thyroid peripheral homeostasis. Masetti et al. conducted a randomized controlled study on mouse models of GD. They studied the gut microbiota, observing a difference in biodiversity, spatial organization, and amount between the THSR immunized treated group and the untreated control group. The THSR immunized group developed signs of ophthalmopathy and their gut microbiota had more Firmicutes and less Bacteroides when comparing controls (68). However, already in 1988, Penhale and Young (69) noticed that modulation of the intestinal microbiota, in murine models affected by autoimmune thyroiditis, consistently made them more or less sensitive to thyroid autoimmunity. Lately, Köhling et al., through use of a PCR-denaturing gradient gel electrophoresis with universal primers targeting V3 region of the 16S rRNA gene and quantitative real-time PCR, showed, in subjects affected by hyperthyroidism when compared with hypothyroid patients, an important difference of the intestinal microbiota composition, especially an overgrowth of bacteria in the small intestine, assessed by breath test with hydrogen glucose (70). Ishaq et al. found that there is a significant disparity between the gastrointestinal microbiota of patients affected by HT and healthy controls (p < 0.05). Particularly, they showed raised level of *Actinobacteria* in HT group as compared to control. Moreover, levels of *Prevotellaceae* and *Veillonellaceae* were lower in diseased group if compared with healthy control, as well as *Bifidobacterium* and *Lactobacillus*; *Veillonellaceae* are commensal bacteria with an important role in regulation of adaptive immunity. At last, *Enterobacteriaceae* and *Alcaligenaceae* were higher in HT group as compared to control. *Shigella* and *Escherichia* can cause a broad spectrum of severe infectious diseases from hemorrhagic colitis to septicemia. In this paper, there’s a clear demarcation of intestinal microbiota texture between HT patients and healthy group. The authors hypothesize that the raised levels of autoantibodies in these subpopulations might modify the structure of gastrointestinal microbiota, but other studies are obviously necessary to comprehend the underlying pathogenesis (71). Zhao F. et al. found similar and various concentrations of bacterial in the bowel microbiome of patients suffering from HT and controls (p = 0.11). This study showed that levels of *Blautia*, *Roseburia*, *Ruminococcus_torques_group*, *Romboutsia*, *Dorea*, *Fusicatenibacter*, and *Eubacterium_hallii_*group were higher in patients affected by HT, while *Fecalibacterium*, *Bacteroides*, *Prevotella_9*, and *Lachnoclostridium* genera were lower in the same patients. Additionally, through the LEfSe method the authors were able to show many differences in these 27 genera composition between the patients and the healthy control, differences that were also correlated with clinical manifestation and laboratory data (72). Both in HT and GD the first therapy strategy is represented by the recovery of euthyroidism, in the first case by hormone replacement therapy, in the second case trough specific antithyroid drugs (73). The achievement of therapeutic goal may significantly differ, in both conditions, from patients to patients. Many factors can have a role: from age, sex, changes in BMI, to other conditions such as the thyroid hormones levels and the cause itself of the hypothyroidism, or the contemporary assumption of other drugs, some sorts of food and drink, soy protein, gastrointestinal malabsorption diseases and infections, bariatric surgery, atrophic gastritis, cystic fibrosis (74). All these parallel situations may influence the dosage of L-T4 replacement therapy in case of hypothyroidism and the severity of hyperthyroidism conditions in case of GD patients under antithyroid treatments. A disadvantage of antithyroid therapy is the high frequency of disease recurrence after the drug has been interrupted; these events occur often in the first year after discontinuing therapy, mostly in the first 6 months (75). In literature we aren’t able to find many evidences about a possible correlation between gut microbiota changes and response to drug therapy. Actual studies have improved our knowledge about human microbiota, its basic functions and dynamics. The characterization of specific subset of bacterial species in ATD could help to identify new therapeutic strategy, by using probiotics, to rapidly gain therapeutic goal. Probiotics are available in many formulations, i.e. in the form of fermentable food, powders, or liquid drops. Many evidences show that probiotics, when given in the just dosage and for an adequate period of time, may lead to beneficial effects to human health; moreover they are often safer than many drugs. Probiotic impact on the immunity is important to comprehend how to therapeutically approach may interfere to the global increasing incidence of autoimmune diseases. INDIGO performed a double-blind, placebo-controlled, randomized clinical study on the effects of a LAB4 probiotic on gut microbiota composition in patients with GD. There was a significant reduction in the Firmicutes phylum count in the treated group compared to placebo (P = 0.033) and a temporary although important reduction in circulating autoantibodies and, consequently, in relapses at 6 months after antithyroid therapy, indicating the systemic immunomodulating effect of probiotics (76).

## Microbiota and Cancer

Some studies have shown the correlation of microbiota with cancer (77). Dysbiosis could initiate inflammatory and pro-carcinogenicity; conversely gut-derived probiotics restore gut commensal bacteria and protect the host including cancer disease, returning to a condition of wellness. Vivarelli et al. found that microbiota interferes directly host’s DNA replication and its integrity. He shows ways of how pathogenic bacteria are capable to promote oncogenesis through the modulation or interfering with specifics host’s oncogenic cell pathway or by interposing either with the hormonal or the host’s immune system. Microbiota changes can release toxins that induce human DNA damage, concurring to its mutability, tumor induction and progression in gastrointestinal cancer. The author continues saying that “gut pathogenic bacteria can also disturb with DNA damage response and repair pathways, as in the case of *Shigella flexneri*, stimulating host’s cells p53 degradation *via* the secretion of its enzymes *inositol phosphate phosphatase D* (IpgD) and *cysteine protease-like virulence gene A*.” Thus, it can increase the risk to induce mutations during the DNA damage response in infected cells. *Cytotoxin associated gene A* from *Helicobacter pylori*, causes the proteasome-mediated degradation of p53 in gastric epithelial cells, by interfering with the host’s AKT pathway and inducing the gastric cancer. It is very complicated to determine in a clear way whether dysbiosis might have effects on the genesis of cancer or not. Additionally, changes in the everyday lifestyle, diet, and immune system factors, which deeply affect the microbiota composition and activity, may affect cancer genesis (78). Currently, Thyroid Carcinoma (TC) is the fifth most common malignancy diagnosed in female gender and its incidence is United States has increased an average of 3% per year in the last 4 decades; this increase is resulted by interactions among environmental factors, lifestyle, and genetic. Bowel microbiota, maybe, is an important environmental factor in gastrointestinal and extraintestinal tumor pathogenesis. However, regarding composition of bowel microbiome in patient affected by TC, there’s limited information. Two categories include the contribution of gut microbiota to tumor pathogenesis. The first category includes the damage to DNA and apoptosis; bacteria like *E. coli* and *Bacteroides fragilis* may influence the host genomic stability leading to DNA damage and mutations, having and important role to colorectal carcinogenesis. The second category includes inflammatory reactions; many pattern recognitions receptors, comprehending *Toll-Like Receptors* (TLRs) are activated by cancer-associated microbial communities stimulating in loop *NF-kB* signaling activation in the tumor microenvironment. Moreover, tumorigenesis obviously is influenced by specific pathogens and metabolic outputs of the gut microbiota. However, it has become increasingly clear that the collective activities of resident gut microbiota, particularly their metabolic products, strongly influence the protection against and predisposition to the development of malignancies. Up to now, there are only two reports in the literature, regarding the characterization of microbiota between healthy subjects, thyroid benign nodules and thyroid cancer patients. Feng et al. evaluated relationships among gastrointestinal microbiota, fecal metabolites, and TC. Through their researches they have noticed significative differences in gastrointestinal microbial communities from patients affected by TC and not affected ones, and also to the high or low quantity of bacteria’s genera in TC, by using Ultra-performance liquid chromatography profiling of fecal samples. In fecal metabolites composition were also identified many differences, indeed 72 metabolites showed important changes. Patients affected by TC were successfully identified from a combination of eight metabolites and five genera. Moreover, the findings implicate alterations in gut microbiota and metabolites in TC pathogenesis (79). In a recent cohort study by Zhang et al., the intestinal microbiota was compared with endocrine thyroid function both in patients with thyroid cancer and in subjects with benign nodular thyroid disease. The results made evident a difference in the microbiota between the thyroid pathology, both benign and malignant, and the healthy population: in particular, it was noticed a significant higher level of *Neisseria* and *Streptococcus* in patients with thyroid diseases while *Butyricimonas* underwent a significant decrease in patients with thyroid cancer and the same happened for *Lactobacillus* in patients with thyroid nodules (p < 0.001). This shows that both pathological situations, benign and malignant thyroid diseases, correlate with the composition of the gut microbiota and consequently poses the possibility of any future treatments with specific probiotics (80). No data are present in the literature about the characterization of intestinal virome in thyroid cancer and thyroid nodules. Data collected over the past decade have identified the gut microbiota as an important factor defining inter individual variation in diseases risk. Evidence shows that gut microbiota and metabolites alter and potentially control cancerogenesis and progression.

## Conclusion

Microbiome-wide association studies have established that numerous diseases are associated with changes in the microbiota. These studies typically generate a long list of commensals implicated as biomarkers of disease, with no clear relevance to disease pathogenesis. A major challenge is to understand which of the many diseases, including inflammatory diseases and various forms of cancer and cancer risk factors are detectable as different locations on this map. Our mini review tried to evaluate the correlation between thyroid diseases and gut dysbiosis, demonstrating that a changing in quality and quantity of intestinal commensal and pathogenic bacteria is associated with development of thyroid endocrine disease such as Hashimoto Thyroiditis and Graves’ Disease as well as thyroid carcinoma. Future investigations are needed to identify the optimal probiotic and dose for specific diseases. The future of these researches is represented by the need to find the strict and direct correlation between the microbial changes and the specific pathology in order to identify the most targeted therapy possible, customized on each patient.

## Author Contributions

All authors contributed significantly to the present research and reviewed the entire manuscript. GDoc: Participated substantially in conception and design of the manuscript and in the analysis and interpretation of the data. AC: Participated substantially in conception, design, and execution of the study and in the analysis and interpretation of the data; also participated substantially in the drafting and editing of the manuscript. RR, MF, CO, VA, MG, GDon, VN, MR, GC and DP: Participated substantially in conception and design of the manuscript and in the analysis and interpretation of the data. All the authors have read an approved the final manuscript.

## Conflict of Interest

The authors declare that the research was conducted in the absence of any commercial or financial relationships that could be construed as a potential conflict of interest.
